# The second docetaxel rechallenge for metastatic castration-resistant prostate cancer: a case report

**DOI:** 10.3389/fonc.2023.1185530

**Published:** 2023-09-27

**Authors:** Wei Ning, Pengkang Chang, Ji Zheng, Fan He

**Affiliations:** ^1^ Department of Urology, Second Affiliated Hospital, Army Medical University, Chongqing, China; ^2^ Urology Department, Institute of Urology (Laboratory of Reconstructive Urology), West China Hospital, Sichuan University, Chengdu, Sichuan, China

**Keywords:** prostate cancer (PCa), metastatic castration-resistant prostate cancer (mCRPC), second docetaxel rechallenge (DR), later-line treatment, case report

## Abstract

**Background:**

Docetaxel combined with prednisone plus androgen deprivation therapy (ADT) is the preferred treatment option for metastatic hormone-sensitive prostate cancer (mHSPC) or metastatic castration-resistant prostate cancer (mCRPC). With the development of next-generation hormonal agents (NHAs) and poly (ADP-ribose) polymerase (PARP) inhibitors, more aggressive first-line or later-line treatment strategies have been added to the treatment of mHSPC and mCRPC. However, docetaxel rechallenge (DR) has special clinical significance in patients with “docetaxel-sensitive” prostate cancer. There are no reports on the efficacy and safety of the second DR in mCRPC patients.

**Case presentation:**

We report one patient diagnosed with mCRPC who showed progression-free survival (PFS) and overall survival (OS) benefits and safety and good lower urinary tract function after the second DR.

**Conclusion:**

The second DR as a potential alternative later-line treatment strategy should be considered for patients with mCRPC who worry about the high economic burden of multigene molecular testing and PARP inhibitors as well as repeated prostate needle biopsy.

## Introduction

The first case of prostate cancer (PCa) was described as a very rare disease by J. Adams at the London Hospital in 1853 (1). Currently, PCa ranks second in the incidence of male cancer and sixth in male cancer mortality worldwide (2). In China, the incidence and mortality of PCa have been rising rapidly for decades. In particular, Chinese patients with PCa have unique epidemiological characteristics, such as higher grading and staging of tumors and a worse disease prognosis (3). Malignant transformation of the normal prostatic epithelium follows a complicated process (4). Metastatic spread of tumors is the main cause of death for patients with PCa. Bone metastases of patients with PCa always manifest as osteoblastic and osteolytic lesions, mainly osteoblastic features, which can lead to severe pain, pathological fractures, hypercalcemia, and nerve compression syndromes (5).

For decades, hormonal therapy, also known as androgen deprivation therapy (ADT), has played an important role in the treatment of patients with advanced PCa and is aimed at lowering testosterone levels. With the development of next-generation hormonal agents (NHAs) and chemotherapy, a more aggressive first-line treatment strategy has been added to the treatment of metastatic hormone-sensitive prostate cancer (mHSPC) (6). Although most patients suffering from mHSPC primarily respond to ADT, the duration of response is uncertain, and all patients ultimately develop metastatic castration-resistant prostate cancer (mCRPC) (7). The standard treatment of mCRPC refers to the combination of ADT and NHA (abiraterone acetate, enzalutamid) or chemotherapy (docetaxel or cabazitaxel). In addition, radium-223, an alpha emitter, can be considered as a treatment for symptomatic bone metastases of patients with PCa (8). Several therapies have been proven to improve the progression-free survival (PFS) and overall survival (OS) of patients with mCRPC. However, most patients eventually die from mCRPC within a few years (7).

Currently, docetaxel is approved for first-line treatment of mHSPC or mCRPC. Reintroduction of docetaxel, which is also known as docetaxel rechallenge (DR), lacks enough supporting evidence in patients with mCRPC (9). The concept of DR represents a special clinical significance in patients with “docetaxel-sensitive” PCa (10). Nevertheless, more high-level evidence is needed for DR as a potential alternative treatment in later lines. Here, we report one mCRPC patient with a second DR as an alternative to poly (ADP-ribose) polymerase (PARP) inhibitors or platinum-based chemotherapy.

## Case presentation

**Figure 1:**
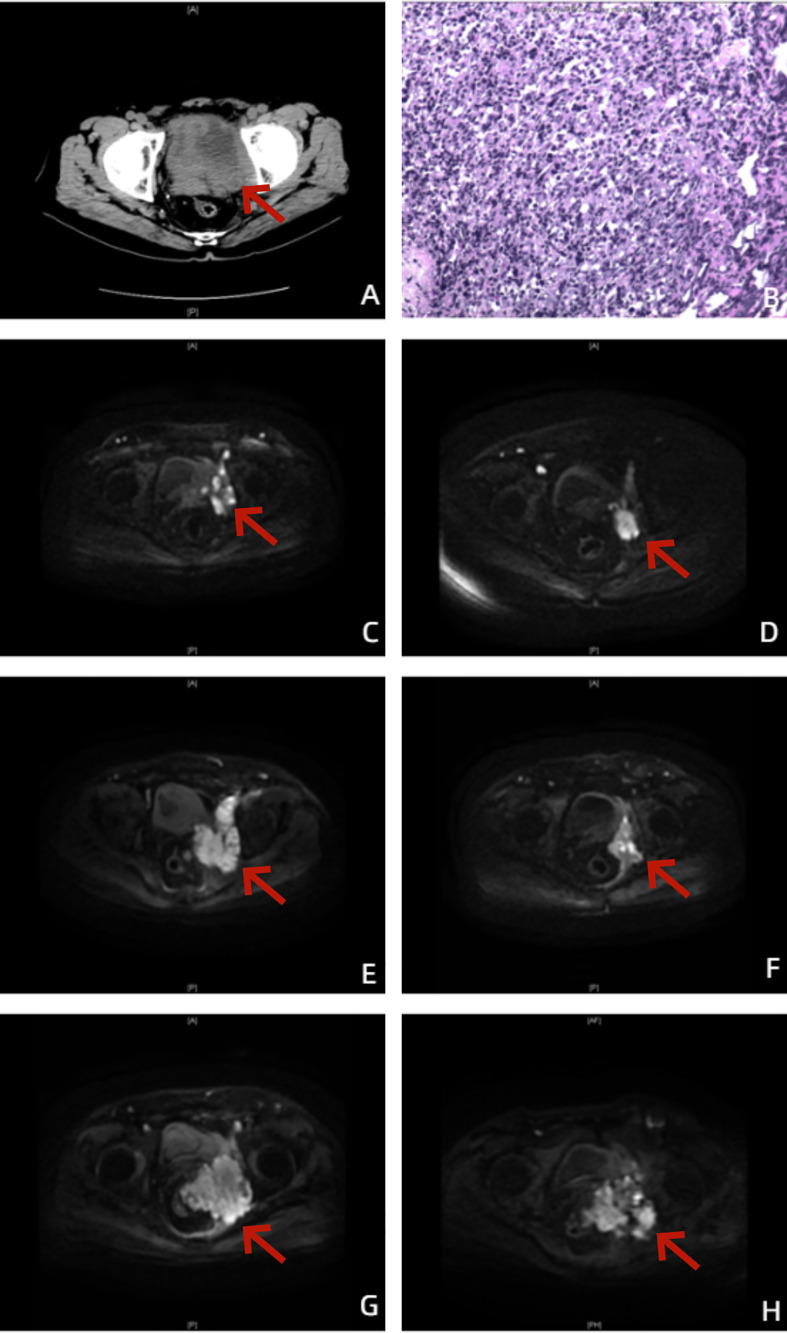
Images of the patient throughout the treatment. **(A)** Before treatment, urinary tract CT showed a localized mass with mixed density in the pelvic region, unclearly displayed prostate and bladder, enlarged pelvic and retroperitoneal lymph nodes, and mildly dilated bilateral renal pelvis and ureter. **(B)** The postoperative pathological diagnosis showed prostate adenocarcinoma, Gleason score (GS) 5 + 4 = 9. **(C)** On 18 June 2020, prostate-enhanced MRI cross-sectional DWI showed an enlarged prostate with a maximum cross-sectional size of 50 × 37 mm, prostate cancer with invasion of the left pelvic sidewall and left side of the bladder, unclearly displayed bilateral seminal vesicles, and enlarged left external and internal iliac lymph nodes. **(D)** On 15 March 2021, prostate-enhanced MRI cross-sectional DWI showed an enlarged prostate with a maximum cross-sectional size of 50 × 37 mm and a significantly reduced volume of the prostate and left pelvic sidewall lesions compared to before treatment. **(E)** On 24 May 2021, prostate-enhanced MRI cross-sectional DWI showed a significantly increased lesion volume in the left pelvic sidewall and new metastasis in the left femoral neck, sacrum, and bilateral iliac crest. **(F)** On 17 November 2021, prostate-enhanced MRI cross-sectional DWI showed prostate with a maximum cross-sectional size of 37 × 29 mm and significantly reduced volume of lesion of prostate and left pelvic sidewall. Except for the left femoral neck, no bone metastases were found in the other parts of the body. **(G)** On 23 March 2022, prostate-enhanced MRI cross-sectional DWI showed that the volume of the lesion of the left pelvic sidewall was significantly increased, and rectal invasion was not ruled out. **(H)** On 8 September 2022, prostate-enhanced MRI cross-sectional DWI did not show new confirmed progression of imaging.

A 70-year-old man was admitted to the Second Affiliated Hospital, Army Medical University on 27 December 2019, with the chief complaint of pollakiuria and urgent urination for half a year and dysuria for 13 days. The patient had urinary incontinence, nocturia, and intermittent hematuria but did not have other clinical symptoms or signs. The patient received transurethral resection of the prostate (TURP) at the local hospital 6 months prior, and the postoperative pathological diagnosis was uncertain. Urinary tract computed tomography (CT) on 14 December 2019, revealed a mass with mixed density in the pelvic region, unclearly displayed prostate and bladder, enlarged pelvic and retroperitoneal lymph nodes, and mildly dilated bilateral renal pelvis and ureter (**Figure 1A**). The prostate-specific antigen (PSA) level of the patient was 198 ng/mL. An enlarged prostate and a hard enlarged mass were palpated by digital rectal examination (DRE). The personal history, family history, and physical examination of the patient were not exceptional.

On 30 December 2019, the patient underwent TURP plus transperineal biopsy of the prostate, and invasion of the left wall of the bladder was observed during the operation. The postoperative pathological diagnosis showed prostate adenocarcinoma, Gleason score (GS) 5 + 4 = 9 (**Figure 1B**).

Positron emission tomography/computed tomography (PET/CT) on 7 January 2020, revealed that the mass in the pelvic region was considered a malignant tumor, and enlarged pelvic and retroperitoneal lymph nodes were considered metastatic carcinoma. The patient was eventually diagnosed with PCa (pT4N1M1a). Because of worrying about the adverse events of docetaxel chemotherapy and the high economic burden of NHA, at the beginning, the patient received ADT (goserelin, 3.6 mg, subcutaneous injection, per 28 days plus bicalutamide 50 mg, oral administration, once daily). To his disappointment, the reduction in the PSA level was unsatisfactory, dropping to only 7.04 ng/mL. Prostate-enhanced magnetic resonance imaging (MRI) on 18 June 2020 revealed an enlarged prostate with a maximum cross-sectional size of 50 × 37 mm, PCa with invasion of the left pelvic sidewall and left side of the bladder, unclearly displayed bilateral seminal vesicles, and enlarged left external and internal iliac lymph nodes (**Figure 1C**). According to the official definition of mCRPC by the European Association of Urology (EAU) guideline as well as Response Evaluation Criteria in Solid Tumors (RECIST) (11, 12), the patient showed radiological progression (an enlarged soft tissue lesion using RECIST), while the PSA level was more than three times as high as 2 ng/mL. The patient has become resistant to ADT with progression to mCRPC, despite serum testosterone remaining at castrate levels (< 50 ng/dL).

From 18 June to 23 October 2020, the patient accepted and started six cycles of docetaxel (75 mg/m^2^, Day 1, intravenous injection, every 21 days) combined with prednisone (5 mg, oral administration, twice daily) plus goserelin. After six cycles of chemotherapy, the PSA level of the patient dropped to 0.97 ng/mL. Prostate-enhanced MRI on 15 March 2021, suggested an enlarged prostate with a maximum cross-sectional size of 50 ×3 7 mm and a significantly reduced volume of the prostate and left pelvic sidewall lesions compared to before treatment (**Figure 1D**). The patient had good lower urinary tract function and clinical efficacy and safety. However, he was still afraid of the adverse events of docetaxel chemotherapy and refused to accept chemotherapy sequentially. After 6 months of follow-up and maintenance ADT, the PSA level of the patient rose to 19.48 ng/mL. Prostate-enhanced MRI on 24 May 2021 revealed a significantly increased lesion volume in the left pelvic sidewall and new metastasis in the left femoral neck, sacrum, and bilateral iliac crest (**Figure 1E**).

We further analyzed the homologous recombination repair (HRR) gene panel of the patient by genetic testing of circulating tumor DNA (ctDNA), and the presence of HRR gene mutations (HRRm) was not found. Therefore, from 25 May to 7 December 2021, the patient accepted and started 10 cycles of docetaxel combined with prednisone (the first DR) plus goserelin and the addition of abiraterone acetate (1,000 mg, oral administration, once daily). Prostate-enhanced MRI on 17 November 2021 revealed a prostate with a maximum cross-sectional size of 37 × 29 mm and a significantly reduced lesion volume in the prostate and left pelvic sidewall. Except for the left femoral neck, no bone metastases were found in the other parts of the body (**Figure 1F**). After 10 cycles of chemotherapy, the PSA level of the patient dropped to 0.61 ng/mL again. Unfortunately, after less than 3 months of follow-up and maintenance goserelin plus abiraterone combined with prednisone, the PSA level of the patient rose to 21.78 ng/mL. In addition, prostate-enhanced MRI on 23 March 2022 revealed that the volume of the lesion on the left pelvic sidewall was significantly increased, and rectal invasion was not ruled out (**Figure 1G**).

**Figure 2:**
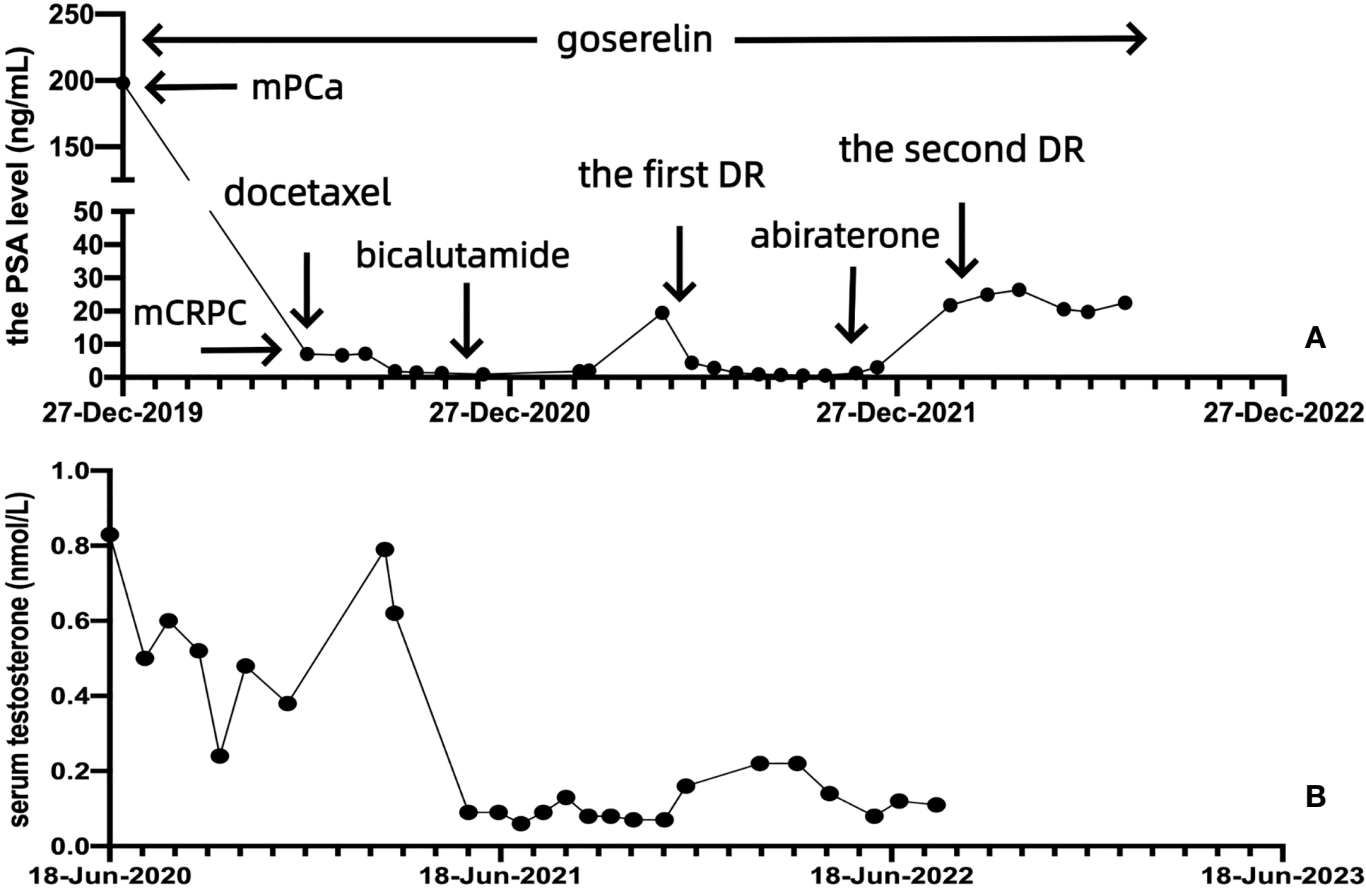
The PSA and serum testosterone levels of the patient throughout the treatment. **(A)** Change in the PSA level after docetaxel chemotherapy, the first DR, and the second DR. **(B)** Change in the serum testosterone level.

After multidisciplinary team (MDT) discussion regarding the worries about the high economic burden of multigene molecular testing by tissue biopsy and PARP inhibitors as well as repeated prostate needle biopsy, on 30 March 2022, the patient accepted and started eight cycles of docetaxel combined with prednisone (the second DR) as well as maintenance goserelin plus abiraterone. Although the PSA response cannot reach a 97% PSA reduction as the first DR, the PSA level of the patient can still be maintained at 19–27 ng/mL. Full- body bone scan and prostate-enhanced MRI (**Figure 1H**) of follow-up did not show new confirmed progression of imaging. The changes in the PSA and serum testosterone levels throughout the treatment are shown in **Figure 2**.

## Discussion

Currently, metastatic PCa (mPCa) remains incurable worldwide. Docetaxel was the first systemic therapy showing a survival benefit to patients with mHSPC or mCRPC (13). Before NHA became available in clinical practice, several studies showed the clinical efficacy of DR in selected patients with mCRPC (14). DR provided moderate clinical efficacy and a maximum PSA response rate of 48%, especially in patients with good PSA responses to first-line treatment with docetaxel.

Because of radiographic progress after ADT, the patient had become resistant to ADT with progression to mCRPC. According to the guidelines, the first-line treatment of docetaxel was administered to the patient with mCRPC, including six cycles of docetaxel combined with prednisone plus goserelin. The PSA level of the patient dropped to 0.97 ng/mL. Prostate-enhanced MRI suggested that the volume of the prostate and left pelvic sidewall lesions was significantly reduced compared to that before treatment. After 6 months of follow-up and maintenance ADT, the PSA level of the patient rose to 19.48 ng/mL. Metastasis of the left femoral neck, sacrum, and bilateral iliac crest was revealed by prostate-enhanced MRI at follow-up.

The E3805 CHAARTED trial revealed significant differences in the transcriptional profile of patients with mPCa, including luminal B subtype, basal subtype, lower androgen receptor activity (AR-A), and high Decipher risk disease. Patients with the luminal B subtype showed a significant OS benefit from ADT + docetaxel (HR 0.45, *p* = 0.007), whereas patients with the basal subtype showed no OS benefit (HR 0.85, *p* = 0.58). Lower AR-A and high Decipher risk were significantly related to poorer prognosis. In addition, patients with high Decipher risk had greater OS improvement from ADT + Docetaxel (HR 0.41, *p* = 0.015) (15). There was a retrospective study of 270 mCRPC patients with good response to first-line docetaxel. The median progression-free interval (PFI) was 6 months from the last chemotherapy of docetaxel. When it recurred, 223 patients received DR, and 47 received other therapy. The median OS for DR and other therapies was 18.2 vs. 16.8, respectively (*p* = 0.35). However, over 6 months of PFI indicated longer OS. Moreover, a good PSA response was more distinct on DR (40.4% vs. 10.6%, *p* < 0.001) (16). Another study showed that DR had OS improvement and safety in patients with a good response to docetaxel initially and more than 3 months of PFI (16). In addition, DR did not seem to increase the risk of adverse events, especially grade 3–4 events (14, 17). However, the GETUG-AFU 15 Phase 3 Trial suggested that only a limited number of patients who received first-line treatment with ADT + docetaxel for mHSPC benefited from DR at mCRPC. At this stage, NHAs such as abiraterone or enzalutamide can be used as a later-line treatment strategy (18). Because of the failure of HRRm testing, the patient received the first DR plus goserelin along with the addition of abiraterone acetate. The PSA level of the patient dropped to 0.61 ng/mL again. The volume of lesions of the prostate and left pelvic sidewall was significantly reduced by prostate-enhanced MRI. Except for the left femoral neck, no bone metastases were found in the other parts of the body. Unfortunately, after less than 3 months of follow-up and maintenance goserelin plus abiraterone, the PSA level of the patient rose to 21.78 ng/mL. In addition, the volume of the lesion of the left pelvic sidewall was significantly increased, and rectal invasion was not ruled out.

In the management of patients with mCRPC, regular MDT discussions can provide a more valuable and individualized treatment strategy, while patients can gain a more prolonged OS and better prognosis (19). After MDT discussion, because of the high economic burden of multigene molecular testing by tissue biopsy and PARP inhibitors, the patient could not receive the combination treatment of olaparib and abiraterone. Moreover, the patient refused prostate needle biopsy again; in turn, he could not confirm the pathological type of neuroendocrine prostate cancer (NEPC) and used platinum-based chemotherapy (20). PCa is a significantly increasing cause of mortality around the world and can also bring about a significantly increasing social and economic burden in modern society. FIRSTANA suggested that the median OS and PFS of patients with mCRPC were 24.3 months and 5.3 months, respectively, with docetaxel combined with prednisone (21). In the end, the patient received eight cycles of docetaxel combined with prednisone (the second DR) as well as maintenance goserelin plus abiraterone. To our excitement, although the PSA response cannot reach a 97% PSA reduction as the first DR, the PSA level of the patient can still be maintained at 19–27 ng/mL. Full- body bone scan and prostate-enhanced MRI during follow-up did not show new confirmed progression on imaging. More importantly, after a total of 24 cycles of docetaxel, the patient was still well-tolerated.

## Conclusion

Overall, we demonstrated that the second DR was associated with further prolonged OS and PFS in patients with mCRPC. The PSA level, MRI progression of the lesion, and adverse events of the patient did not increase significantly. Therefore, this result can be added to the later-line treatment strategy of patients with mCRPC. In the future, more patients who worry about the high economic burden of testing and treatment as well as repeated prostate needle biopsy are advised to consider this later-line treatment strategy. We also believe that this strategy should be popularized by urological clinicians in hospitals.

## Data availability statement

The raw data supporting the conclusions of this article will be made available by the authors, without undue reservation.

## Ethics statement

The studies involving humans were approved by Ethics Committee of Second Affiliated Hospital, Army Medical University. The studies were conducted in accordance with the local legislation and institutional requirements. The participants provided their written informed consent to participate in this study. Written informed consent was obtained from the individual(s) for the publication of any potentially identifiable images or data included in this article. Written informed consent was obtained from the participant/patient(s) for the publication of this case report.

## Author contributions

FH and JZ revised the manuscript. WN was responsible for data collection, data analysis, data interpretation, and writing the manuscript. PC was responsible for image design. All authors contributed to the article and approved the submitted version.
